# Impact of noise on theta-nested gamma oscillations in a spiking continuous attractor model of grid cells

**DOI:** 10.1186/1471-2202-14-S1-P295

**Published:** 2013-07-08

**Authors:** Lukas Solanka, Mark CW van Rossum, Matthew F Nolan

**Affiliations:** 1 Doctoral Training Centre in Neuroinformatics and Computationial Neuroscience, University of Edinburgh, Edinburgh, EH8 9AB, UK; 2 Institute for Adaptive and Neural Computation, University of Edinburgh, Edinburgh, EH8 9AB, UK; 3 Centre for Integrative Physiology, University of Edinburgh, Edinburgh, EH8 9XD, UK

## 

Grid cells in the medial entorhinal cortex (MEC) encode location through firing fields that form grid-like maps of the environment. At the same time network activity in the MEC is dominated by oscillations in the theta (4-12 Hz) and gamma (30-100 Hz) bands. Our recent experimental data established that feedback inhibition between excitatory stellate cells and inhibitory fast spiking interneurons dominates the synaptic connectivity in layer II of the MEC, and that continuous attractor models derived from these properties are sufficient to explain both the network oscillations and grid firing fields [1]. Here we use a spiking continuous attractor network model of layer II stellate cells and fast spiking interneurons to examine the effect of intrinsic noise on the emergence of theta nested-gamma oscillations. In this model, stellate cells connect exclusively to interneurons, while interneurons contact only stellate cells. When driven with a theta (8 Hz) modulated external input, we observed network synchronization in the gamma range during the trough of the theta signal, which depended on the amount of intrinsic Gaussian noise generated independently at each neuron. Without noise, the network exhibited aberrant hyper-synchronous firing of a subpopulation of stellate cells at the beginning of each theta cycle. The stellate cell firing resulted in a strong feedback inhibitory postsynaptic currents from fast spiking interneurons that had an additional synchronizing effect. The strong inhibitory feedback abolished nested gamma oscillations. With increasing noise, the network entered an intermediate state in which we observed theta nested-gamma oscillations [1]. When noise level was set even higher, the nested gamma oscillations disappeared without increasing the amount of inhibitory coupling. We also present the impact of noise on bump attractor formation and gridness score of the grid-like receptive fields. Together, these results show that the presence of noise may have profound implications on the stability of theta-nested gamma oscillations in attractor neural circuits with feedback inhibition.
